# The feeling of throwing good money after bad: The role of affective reaction in the sunk-cost fallacy

**DOI:** 10.1371/journal.pone.0209900

**Published:** 2019-01-08

**Authors:** Koen A. Dijkstra, Ying-yi Hong

**Affiliations:** Department of Marketing, CUHK Business School, Shatin, Hong Kong; Middlesex University, UNITED KINGDOM

## Abstract

Continuing investing in a failing plan (i.e., the sunk-cost fallacy) is a common error that people are inclined to make when making decisions. It is impossible to get resources back that already have been invested. Hence, economic theory implies that decision makers’ decisions should only be guided by future gains and losses. According to the literature, the sunk-cost fallacy is driven by negative affect. Previous studies focused on negative incidental affect. We investigated, in contrast, whether the sunk-cost fallacy is caused by integral affect elicited by the specific decision context. Study 1 demonstrated a positive relationship between affective reaction and the sunk-cost fallacy. Study 2 replicated the finding in Study 1 in a within-subjects design, and demonstrated a full mediation of type of scenario (invest vs. non-invest) on the sunk-cost effect, mediated by integral affective reaction. A mediation using a within-subjects design additionally demonstrated that the effect is mediated by integral emotional responses experienced in relation to each scenario, and not by incidental emotional states that are unrelated to the scenarios. Study 3 replicated findings in the previous studies, and demonstrated that the relation between the sunk-cost fallacy and affect is moderated by justification. Participants who justified their decision were more resistant to the sunk-cost fallacy, and showed less negative affect elicited by the scenarios, than participants who did not justify their decision. Study 4 provided supporting evidence for our hypothesis by hindering conscious deliberation, and promoting reliance on affect, via cognitive load. The results showed that the relation between affect and the sunk-cost fallacy was stronger for participants under high cognitive load, than under low-load. The paper discussed how this research leads to new ways to protect against the sunk-cost fallacy in the discussion.

## Introduction

You ordered a full course dinner at a restaurant that includes several appetizers, entrée, main course and dessert. All the courses were very good and now you are eating your dessert; but after having a few bites of your dessert, you feel satiated and would rather not eat any more of it. What would you most likely do? Stop eating, or continue trying to finish the dessert? Many people will continue eating the dessert in order to justify earlier investments that are already made. That is, having ordered the full course dinner and need to pay for it, people feel they should finish it, even though continue eating is not enjoyable anymore; it would be a waste to “pay and not eat”. The same can occur in other domains; such as watching a boring movie on pay TV you already paid for, or continue working on a project related to one of your hobbies you are bored with. Not watching, or quit working, would both be a waste of invested resources—money paid for pay TV, and hours invested in the project. However, it is impossible to get the resources back that you have invested already. Economic theory implies that decision makers’ decisions should only be guided by future gains and losses, as prior costs do not affect the objective outcomes of current decisions. Hence, the normative correct decision in sunk-cost situations is to ignore past investments. Taking into account past losses or investments is a decision strategy that has been dubbed the 'Sunk-Cost fallacy' or 'Sunk-Cost effect'. It is considered a mistake or faulty strategy. In more neutral terms, as Arkes and Blumer ([1] p. 124) put it, the sunk-cost effect refers to the tendency “to continue an endeavor once an investment in money, effort, or time has been made”.

Experimental evidence and theorizing suggested that the sunk-cost effect is driven by negative feelings caused by the prospect of having invested without success, in other words, having lost investments. After having incurred sunk-costs, people are prepared to take the risk of further losses that continuing investing may bring [2, 1, 3–7]; see also [8]. Arkes [6] argued that the sunk-cost effect is motivated by ‘not to waste’. Researchers theorized that thinking about irretrievable investments causes the elicitation of negative affect that would consequently encourage investment in a failing plan. For example, Bruine de Bruin, Strough, and Parker [9] explained differences in susceptibility to the sunk-cost effect between young and elderly adults by the positivity bias of older, and negativity bias of young adults. Older adults would focus less on negative information than young adults do, and consequently show a lower susceptibility to the sunk-cost fallacy, than young adults. Similarly, Van Putten, Zeelenberg & van Dijk [10] refer to negative affect to explain the relation between state orientation and the sunk-cost fallacy.

Direct evidence for the relation between affect and the sunk-cost fallacy is provided by Moon, and colleagues [11]. They showed a positive correlation between anxiety and employment of the sunk-cost fallacy, and a negative correlation between depression and committing the fallacy. The authors argued that anxious individuals might be more sensitive to pressures embedded within sunk-cost situations, and are therefore motivated to continue investing in a failing plan. As the sunk-cost fallacy is fueled by unrealistically positive future expectations, individuals who suffer from depression are more likely to see their environment as being out of their control, and stop investing additional resources (depressed individuals consistently underestimate their chances of success relative to non-depressed people [12].

Wong, Yik, and Kwong [13] examined how trait neuroticism and personal responsibility interacted in the sunk-cost effect. In their contribution, they pitted three perspectives against each other (i.e. coping, depressive realism, and cognitive dissonance), as each perspective leads to competing hypotheses. The results of three studies supported the coping perspective; people high on neuroticism tend to avoid a stressful situation by engaging in an alternative task rather than the task that is responsible for the stressful experience (i.e. stop continuing investing). This effect was limited to situations where people are personally responsible, the correlation disappeared in conditions where people are not responsible for the situation. People low on neuroticism, in contrast, are less likely to stop continuing investing. They experience lower levels of stress, and correspondingly experience the incentive to change behavior to a lower extent, than people high on neuroticism.

Furthermore, Coleman [14] showed experimental evidence for a larger sunk-cost effect for participants in which anger was induced than participants who were induced to feel fear. Hafenbrack, Kinias, & Barsade [15] demonstrated, among other findings, that the sunk-cost effect was attenuated by reducing state negative affect through mindfulness meditation.

Wong & Kwong [16] studied *anticipated* regret and demonstrated that the sunk-cost effect is stronger when the possibility of future regret about withdrawal of, or continuing, commitment is high than when this possibility is low. Ku [17], in contrast, demonstrated that decision-makers can learn from escalating situations; he showed that *post-escalation* regret experienced in another task reduced subsequent escalation in a sunk-cost situation in question, with more regret predicting less escalation. The same effect was found for decision-makers who were instructed to imagine an escalation situation beforehand.

### Integral and incidental affect

Research showed that behavior is at least partly affected by the current affective reaction [18]; (see also [19]); for the role of emotion in judgments of cooperation (see [20]). This affective reaction can be elicited by sources of information that are unrelated or irrelevant to the judgment or decision in question, such as affect elicited by the environment (e.g., [21]). This so-called Incidental affect encompasses all factors that elicit affect, but are unrelated to the judgmental target (e.g., mood, priming, motor affect, affective conditioning; [22]). Mood states, for instance, may be used as a heuristic when making evaluative judgments [18]. According to the mood-as-information heuristic people make evaluative judgments by asking themselves “How do I feel about it?”, consequently they will monitor their own feelings, and make judgments congruent with their own mood [23]. When people are notified of the cause for their feelings, the effect of the mood-as-information heuristic disappears (e.g., [24])

*Integral affect*, on the other hand, is part of the perceiver’s internal representation of the option or target under consideration. According to Damasio [25], images become “marked” by positive and negative feelings linked to somatic or bodily states, by a lifetime of learning. When a negative somatic marker is linked to an image of a future outcome, it sounds an alarm. When a positive marker is associated with the outcome image, it becomes a beacon of incentive. Damasio argued that these signals increase the accuracy and efficiency of the decision-making process, and that without these feelings, information lacks meaning.

Slovic, Finucane, Peters and Macgror [26] expanded on this idea in their affect heuristic. Similar to the somatic marker hypothesis, the affect heuristic posits that representations of objects and events in people’s mind are tagged to varying degrees with affect. Whenever considering or confronted with a representation or object, the first reaction is an automatic affective evaluation [27]. According to the affect heuristic, this automatic evaluation is done by consulting the “affect pool”. This affect pool contains all the conscious and unconscious positive and negative tags associated with the representation or object and serves as a cue for judgment and decisions. Using this overall, readily available affective impression is easier and more efficient than weighing pros and cons or retrieving experiences or examples from memory. Especially when the required judgment or decision is complex or mental resources are limited.

### The present research

Previous studies that investigated mechanisms of affect in sunk-cost situations (mentioned above) either used general trait affect (e.g., personality trait anxiety), or induced emotions that were not related to the sunk-cost scenario (e.g., anger in a study by Coleman [14]), both are incidental emotional states. The purpose of the present research is to investigate a closer link between ‘integral’ affective responses and the sunk-cost fallacy by measuring affective reaction caused directly by each sunk-cost scenario.

Study 1 investigated the relation between affect elicited by sunk-cost scenarios by directly measuring affect after each fictional scenario. Next, Study 2 replicated the findings in Study 1 within a repeated measurements design using sunk-cost scenarios in which an investment has been done, and in their non-, or very small, investment equivalent.

To investigate psychological effects of waste aversion, and its consequent effect on willingness to invest in sunk-cost scenarios, together with its effect on the resulting experience of affect, we induced participants to justify their responses vs. no-justification in Study 3. Cognitive load was induced in Study 4 to provide supporting evidence for our hypothesis that affect is a driving force in the sunk-cost fallacy.

Across all studies, we disclose all measures, manipulations, and exclusions, as well as the method of determining the final sample size. Ethical approval for the studies was provided by the Chinese University of Hong Kong Survey and Behavioural Research Ethics Committee (SBREC), for all four studies. All subjects provided written informed consent prior to participating.

## Study 1

Study 1 investigated the relation between the affective reaction elicited by sunk-cost scenarios, and participant’s subsequent decision. Because affect (i.e., incidental emotional states) has been linked to the sunk-cost fallacy by others (see above), we hypothesized that the affective reaction elicited by a particular scenario (i.e., integral emotional responses) to be related to employment of the sunk-cost fallacy. Directly linking the experience of affect to the scenario under consideration (not affect experienced that is unrelated to the scenario, such as mood), would provide direct evidence of its role in investment choices in the scenario in question.

### Method

#### Participants

Eighty-nine students at the Chinese University of Hong Kong participated in the study. Age ranged from 17 till 27 years old (*M* = 20.79, *SD* = 2.27); 35 participants were male and 54 were female (An a priori power analysis revealed that at least 82 participants are required to detect a medium sized effect of *ρ* = .3 with a statistical power of .80, within a Bivariate model).

#### Materials and procedure

Participants responded to 10 scenarios that described a sunk-cost situation ([28] see appendix A; The 10 scenarios used in the study can be categorized into two types of investment; financial and effort. Comparing sunk-cost scores between the two types of scenarios revealed that investments that involve a financial investment are more susceptible to the sunk-cost fallacy (i.e., lower scores; sunk-cost score: M = 3.84, SD = .83) than scenarios that involve an investment in terms of effort (sunk-cost score: M = 4.58, SD = .88), F(1,85) = 35.77, p < .001, η_p_^2^ = .30. The intention of Bruine de Bruin et al. [28] was to calculate an aggregated score and thus the results based on sub-categories of scenarios will need replication in future studies). After the description of each scenario, valence of the affective response was assessed using the 11-point pleasure scale by Lang, Bradley, and Cuthbert [29]. This scale was anchored from 1, very unhappy, to 11, very happy. To make sure that participants interpreted the pleasure scale in the intended way, we explained the scale and anchors, using the instruction by Lang et al. [29]. Specifically, the participants were asked: “How did you feel when deciding how to react in the problem?”. The lower the score, the more negative the emotion.

After indicating their affective response elicited by each scenario, participants were asked to select the option on a scale ranging from 1 (most likely to choose [the sunk-cost option]) through 6 (most likely to choose [the normatively correct option]) that best reflected their relative preference between the two options, in response to the scenario. The lower the score, the stronger preference for sunk-cost options.

### Results and discussion

Three participants were excluded from the analyses because the mean response on the pleasure scale deviated more than 2.5 standard deviations from the sample mean response. No responses deviated more than 2.5 standard deviations on the scale that measured sunk-cost.

Mean valence of affective reaction was *M* = 4.56, *SD* = .85. Mean response on the sunk-cost scale was *M* = 4.14, *SD* = .64. Both scales were positively correlated (*r* = .27, *p* = .013, η_p_^2^ = .07). Negative affective responses to scenarios were related to higher sunk-costs decisions.

## Study 2

Study 2 aimed to conceptually replicate the findings in Study 1 using a within-subjects design. In addition, the study aimed to test a mediation effect of scenario type (i.e., invest vs. non-invest scenarios) on the sunk-cost effect, by affective reaction. A mediation by affective reaction would also demonstrate that the effect is mediated by integral emotional responses experienced in relation to each scenario, and not by incidental emotional states that are unrelated to the scenarios.

### Method

#### Participants

We recruited 60 participants (26 male and 34 female) online using Amazon’s Mechanical Turk (www.mturk.com; [30–33]). The study was approved by the Chinese University of Hong Kong Survey and Behavioural Research Ethics Committee (SBREC). All subjects provided written informed consent prior to participating.

Age ranged from 19 to 65 years old (*M* = 38.95, *SD* = 12.55). Behavioral research conducted on participants recruited on Amazon’s Mechanical Turk demonstrated to be able to replicate research findings established in a student population (e.g., [34]). Participation was limited to US residents only (a priori power analysis revealed that at least 64 participants are required to detect a medium sized effect of ρ = .30 within a correlation, using a statistical power of .80).

#### Materials and procedure

Participants were presented with two pairs of scenarios [35] (see appendix B). Each pair consisted of one scenario involving an investment, and an analogous scenario involving no investment. For example, one of the investment scenarios said, ‘‘you paid $10.95 to see a movie on pay TV. After 5 minutes, you are bored and the movie seems pretty bad.” In the non-investment analogue, the sentence about the $10.95 payment was removed. One pair of scenarios involved a monetary investment, and the other involved a time investment; order of type of scenarios was randomized and counterbalanced. Each scenario was separated from the other three scenarios by three filler scenarios. As in Study 1, affective response was assessed using the pleasure scale immediately after reading each scenario.

After indicating their affective response to each scenario, participants selected one of five options for future time investment (e.g., 1: watch until the end, 2: watch for 30 more minutes, 3: watch for 20 more minutes, 4: watch for 10 more minutes, 5: stop watching entirely). The response options for future time investment were similar to Strough et al. (2008) [35]. As such, the lower the score, the stronger the sunk-cost effect.

### Results and discussion

Two participants were excluded from the analyses because the difference on the pleasure scale of the two types of scenarios deviated more than 2.5 standard deviations from the sample mean response. One participant deviated more than 2.5 standard deviations on the difference between the sunk-cost scores in the two types of scenarios, and was excluded.

A repeated measures ANOVA on mean sunk-cost scores for invest and non-invest scenarios revealed that invest scenarios showed lower sunk-cost scores (i.e., stronger sunk-cost effect) (*M* = 3.04, *SD* = 1.03) than non-invest scenarios (*M* = 3.90, *SD* = 1.00), *F*(1, 56) = 38.12, *p* < .001, η_p_^2^ = .41, replicating findings by Strough and colleagues [35–36]. This suggested that the sunk-cost fallacy is elicited by aversion toward investment lost.

Another repeated measure ANOVA on affective reaction in invest and non-invest scenarios revealed that invest scenarios elicited less positive (more negative) affective reactions (M = 4.41, SD = 1.78) than non-invest scenarios (*M* = 5.11, *SD* = 1.68), F(1, 56) = 21.33, *p* < .001, η_p_^2^ = .28.

A within-subjects mediation analysis was conducted to test whether the relationship between invest conditions and the sunk-cost fallacy was mediated by affective reaction elicited by the scenarios. We utilized the MEMORE macro for SPSS [37]) to do so. This method allowed for entering within-subjects data as dependent, and mediator variables; The within-subjects mediation assesses the relationship between the difference scores of the mediators and outcome variable [38].

Supporting our predictions, affective reaction in invest minus non-invest scenarios difference scores predicted the sunk-cost difference scores, *β* = .35, *t*(56) = 2.80, *p* = .007, η_p_^2^ = .13 (Predicting sunk-cost scores by affective reaction in invest scenarios, while controlling for affective reaction and sunk-cost scores in non-invest scenarios, yielded similar results).

Entering sunk-cost scores, and affective reactions for invest, and non-invest, scenarios in the two-condition within-subjects (5.000 bootstrap samples) statistical mediation analysis, revealed that the 95% CIs for the indirect effect of type of scenario on responses on the sunk-cost scale through affective reaction did not contain zero, 95% CI = (-.48, -.04), providing evidence for statistical mediation.

## Study 3

Studies 1 & 2 demonstrated that the sunk-cost fallacy is driven by the negative affective reaction that sunk-cost scenarios elicit. In Study 3, we examined how this finding fits explanations for the sunk-cost fallacy provided in the literature. As mentioned in the Introduction, researchers have theorized that the sunk-cost fallacy is triggered by waste-aversion, or considering irretrievable losses e.g. [6]. We reasoned that forgoing investments (e.g., waste) would cause lower levels of negative affect if forgoing investments are justified, compared to situations when forgoing investments are not justified. In Study 3 we tested affect elicited by sunk-cost scenarios, and susceptibility to the sunk cost fallacy, as a function of justification.

### Method

#### Participants

We recruited 128 participants (51 male and 77 female) online using Amazon’s Mechanical Turk. Age ranged from 18 to 69 years old (*M* = 35.77, *SD* = 11.86). Participation was limited to US residents only (a priori power analysis revealed that at least 128 participants are required to detect a medium sized effect of *f* = .25 within an ANCOVA design, using a statistical power of .80).

#### Materials and procedure

Participants were randomly assigned to the justification and non-justification condition, and completed five scenarios that described a potential sunk-cost situation (selected from the 10 scenarios used in Study 1; see appendix A). Before providing their relative preference between the two courses of action in each scenario, participants responded to the affective reaction measurement, similar to Studies 1 & 2.

Participants in the justification condition were instructed to think for one minute about reasons for their decision in each scenario, note one down, and respond with their relative preference between the two options. Those in the control condition were instructed not to think too much, before providing their judgment.

### Results and discussion

One participant deviated more than 2.5 standard deviations from the sample mean response on the affective scale and is consequently excluded from the analysis. No participants deviated more than 2.5 standard deviations from the sample mean of the sunk-cost scale.

Decisions in the justification condition were more normatively correct (*M* = 4.75, *SD* = .99) than decisions in the non-justification condition (*M* = 4.42, *SD* = .91; *F*(1,125) = 3.93, *p* = .05, η_p_^2^ = .03). Correspondingly, affective reaction in response to the scenarios were less negative in the justification condition (*M* = 6.12, *SD* = 1.59) than in the non-justification condition (*M* = 5.06, *SD* = 1.27, *F*(1,125) = 17.58, *p* < .001, η_p_^2^ = .12). Both results provide evidence that participants felt less bad about irretrievable investments, and were less susceptibility to the sunk cost fallacy, after justification than without justification.

**Fig 1:**
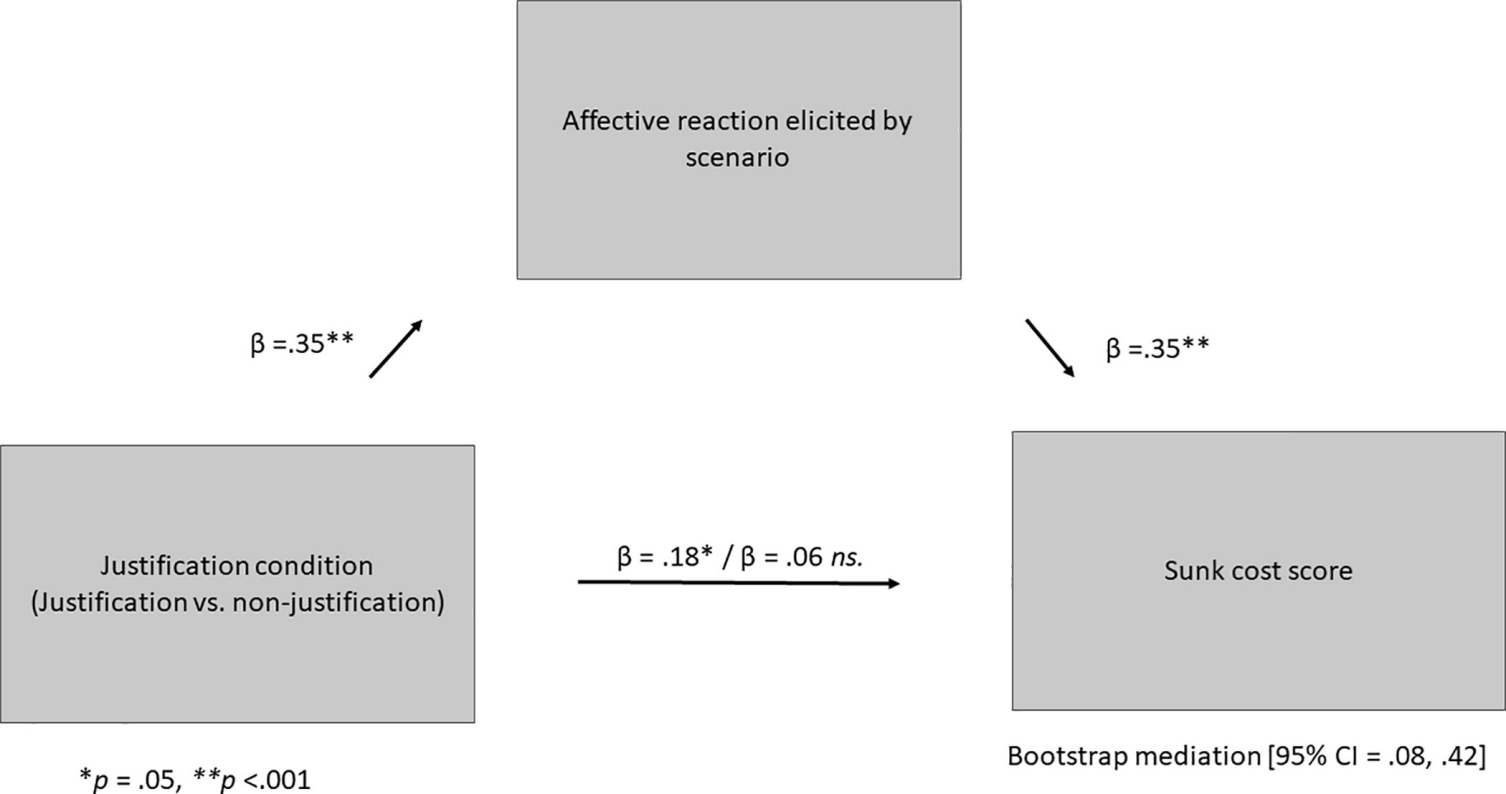
Mediation of the effect of condition (justification vs. non-justification) on sunk-cost scores through affective reaction.

Affective reaction predicted sunk-cost scores in the scenarios, *β* = .35, *t*(125) = 4.13, *p* < .001, η_p_^2^ = .12. Mediation bootstrap analyses were conducted ([39] using 5,000 samples) to calculate confidence intervals (CIs), and tested whether effect of justification on sunk-cost scores is mediated by affective reaction elicited by the scenarios. The analyses revealed that the 95% CI for the indirect effect of condition (justification vs. non-justification) on the sunk-cost scale through affective reaction did not contain zero, 95% CI = .08, .42], providing evidence for statistical mediation, see Fig 1.

## Study 4

To gain insight in the underlying process of the sunk-cost fallacy, its link with affect, and find supporting evidence for the finding in Study 3, we induced cognitive load in Study 4. Cognitive load is expected to prevent participants to make a conscious deliberation in decision-making and promote reliance on affect.

Affective reactions are often the very first reactions that people experience. They occur automatically and can subsequently guide information processing and judgment [27]. Integral affective feelings are also more readily accessible than thoughts when making decisions [40]. Affective reactions might therefore be the last source of information that get interfered by cognitive load. As a result, affective reactions are expected to be especially influential in the high-load condition, compared to the low-load condition. In the latter condition, affective reactions are expected to be less influential because judgments might be affected by more conscious processing.

### Method

#### Participants

We recruited 134 participants (60 male and 74 female) online using Amazon’s Mechanical Turk. Age ranged from 19 to 72 years old (*M* = 39.18, *SD* = 12.03). Participation was limited to US residents only (Similar to Study 3, an a priori power analysis revealed that at least 128 participants are required to detect a medium sized effect of *f* = .25).

#### Materials and procedure

Participants were randomly assigned to the low- and high-load condition, and completed pairs of scenarios, together with the assessment of valence of affective responses after reading each scenario, but before giving their response to the scenarios. The scenarios and assessment of affective responses was the same as in Study 2.

**Fig 2:**
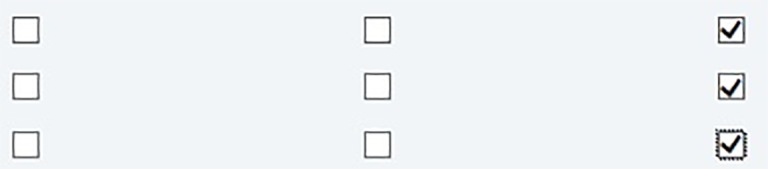
Example of a check marks pattern in the low-load condition.

**Fig 3:**
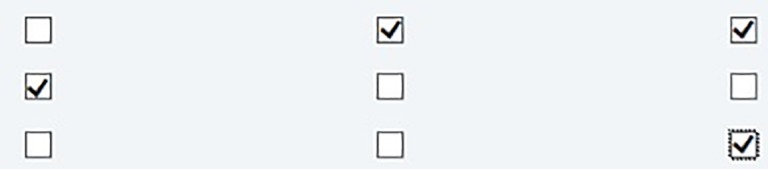
Example of a check marks pattern in the high-load condition.

To manipulate cognitive load, we used an adaption of the Dot Memory Task, a standard spatial storage task that have been used by other authors to induce cognitive load (e.g. [41–45]). Instead of patterns of dots, we used patterns of check marks. Before reading each scenario, participants briefly saw a matrix in which some cells were filled with check marks creating a certain pattern. Participants were instructed to memorize the position of the check marks (which was different every time). Once completing the sunk-cost scenario, and the assessment of affective response, participants had to reproduce the check marks configuration in an empty matrix. Participants in the low-load condition saw very easy 3 x 3 matrices similar to that presented in Fig 2. Participants in the high-load condition saw difficult 3 x 3 matrices similar to that presented in Fig 3. The 3 x 3 matrices were presented for approximately 1 second. We recorded the number of correctly remembered matrixes for each participant.

Participants’ current mood was assessed at the start and end of the experiment to test whether the load induction affected mood.

To recapitulate; the sequence of assessment of response, and affective reaction, to each scenario, together with cognitive load induction in each trial, was as follows. (1) presentation pattern of check marks, (2) presentation of sunk-cost scenario, (3) assessment of affective reaction in response to each scenario, (4) selection of course of action in response to the scenario, (5) replication pattern of check marks.

### Results and discussion

Four participant were excluded from the analyses because the difference on the affective response scale in the two types of scenarios deviated more than 2.5 standard deviations from the sample mean response. Three participants were excluded because the difference on the sunk-cost scale deviated more than 2.5 standard deviations from the sample mean response.

Participants in the low-load condition replicated more matrices correctly (M = .88, SD = .19) than participants in the high-load condition (*M* = .64, *SD* = .31); *F*(1,125) = 26.57, *p* < .001, η_p_^2^ = .18^.^(Removing participants based on number of replicated patterns did not affect the pattern of results in the analyses below). This confirms that remembering easy 3 x 3 patterns was less demanding than remembering difficult 3 x 3 matrices.

Measurement of current mood before (Low load: *M* = 5.37, *SD* = 1.10; High load: *M* = 5.31, *SD* = 1.14) and after the experiment (Low load: *M* = 4.98, *SD* = 1.35; High load: *M* = 4.81, *SD* = 1.31) did not interact with load conditions (*F*(1,125) = .61, *p* = .44, η_p_^2^ = .005). This demonstrated that load conditions did not affect current mood.

A repeated measures ANOVA on mean sunk-cost scores for invest and non-invest scenarios revealed that invest scenarios showed lower sunk-cost scores (i.e., stronger sunk-cost effect) (M = 3.74, SD = 1.25) than non-invest scenarios (*M* = 4.34, *SD* = 1.17), *F*(1, 125) = 35.24, *p* < .001, η_p_^2^ = .22. Another repeated measure ANOVA on affective reaction in invest and non-invest scenarios revealed that invest scenarios showed less positive (more negative) affective reactions in response to the scenarios (*M* = 4.36, *SD* = 1.33) than non-invest scenarios (*M* = 4.72, *SD* = 1.07), *F*(1, 125) = 14.66, *p* < .001, η_p_^2^ = .11.

Decisions in the invest scenarios in the high-load condition were more normatively correct (*M* = 4.01, *SD* = 1.22) than in invest scenarios in the low-load condition (*M* = 3.47, *SD* = 1.24), *F*(1,125) = 6.11, *p* = .015, η_p_^2^ = .05.

A moderating effect of condition for non-invest scenarios was also present; sunk-cost scores in non-invest scenarios were higher in the high-load condition (*M* = 4.55, *SD* = .91) than in non-invest scenarios in the low-load condition (*M* = 4.13, *SD* = 1.37), *F*(1,125) = 4.17, *p* = .043 η_p_^2^ = .03. Both scores indicated that participants showed more normatively correct decisions (i.e. higher sunk-cost scores) in the high-load than in the low-load condition. No effect of condition was present for affective reaction in invest scenarios, *F*(1,125) = 1.50, *p* = .22, η_p_^2^ = .01. Neither was there an effect of condition for affective reaction in non-invest scenarios, *F*(1,125) = 1.53, *p* = .70, η_p_^2^ = .01.

Affective reaction in invest scenarios predicted employment of the sunk-cost fallacy, β = .37, *t*(125) = 4.45, *p* < .001, η_p_^2^ = .14. Moreover, a moderation analysis revealed this effect interacted with cognitive load, *β* = .80, *t*(123) = 2.95, *p* = .004, η_p_^2^ = .07. We conducted simple slope analyses to gain insight in the moderation effect in invest-scenarios. In agreement with our hypothesis the simple slopes analyses revealed that within the high-load condition, affective reaction predicted sunk-cost scores, *β* = .63, *t*(123) = 5.62, *p* < .001, η_p_^2^ = .40. The effect of affective reaction on sunk-cost scores was only marginally significant under low cognitive load, *β* = .21, *t*(123) = 1.90, *p* = .060, η_p_^2^ = .05. Not surprisingly, affective reaction in non-invest scenarios did not predict the tendency to commit the sunk-cost fallacy, *t*(125) = -1.01, *p* = .32, η_p_^2^ = .008. Neither was this test moderated by cognitive load, *t*(123) = 1.08, *p* = .28, η_p_^2^ = .009.

Measurement of mood at the start of the experiment in addition provided the opportunity to test the alternative explanation that the sunk-cost effect is affected by mood instead of affective reaction. This analysis revealed that mood (if any effect) had a reversed trend on the sunk-cost scores, a more positive mood resulted in higher employment of the sunk-cost fallacy, *β* = -.14, *t*(125) = -1.59, *p* = .11, η_p_^2^ = .020.

**Fig 4:**
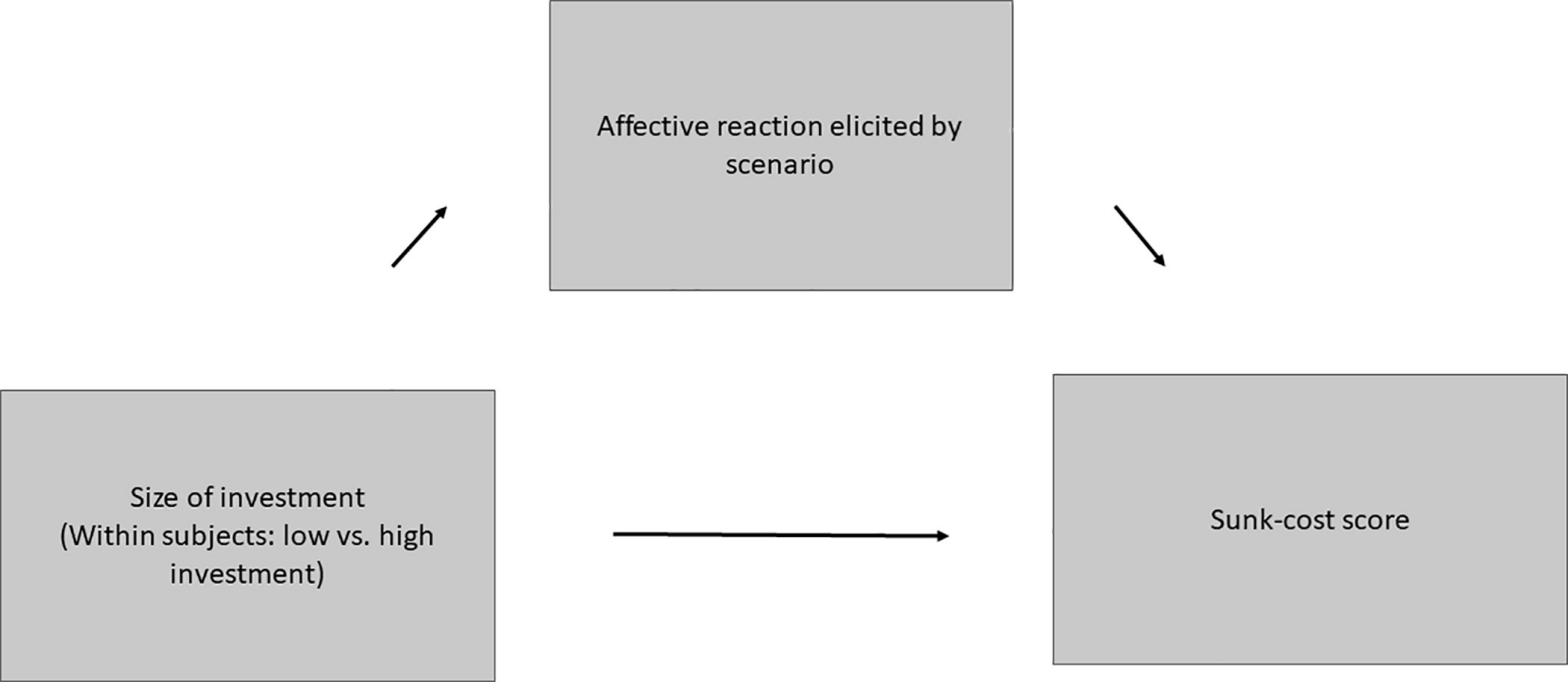
Within-participant mediation model; effect of condition (low vs. high investment) on sunk-cost scores through affective reaction in the high-load condition.

To test for mediation in the high-load condition we subjected sunk-cost, and affective reaction scores for both type of scenarios to a two-condition within-participant, statistical mediation analysis (5.000 bootstrap samples), similar to Study 2 (see Fig 4). This analysis revealed that the 95% CIs for the indirect effect of type of scenario on responses on the sunk-cost scale through affective reaction did not contain zero, 95% CI = (-.48,-.08), providing evidence for statistical mediation. The same analysis for the low-load condition showed that the indirect effect of type of scenario on responses on the sunk-cost scale through affective reaction contained zero, 95% CI = (-.09, .14), indicating that the mediation was not significant in the low-load condition.

## General discussion

In four studies, we showed that the sunk-cost fallacy is related to negative affective reactions directly experienced after, and elicited by, sunk-cost scenarios. Study 1 showed a correlation between the sunk-cost fallacy, and the affective reactions elicited by the sunk-cost scenarios. Study 2 replicated these findings using a within-subjects design, and additionally showed a mediation of the effect of type of scenario (invest vs. non-invest) on sunk-cost scores, by affective reaction. Study 2 also demonstrated that besides incidental emotional states (demonstrated by [11, 14 and 15]), also ‘integral’ affective responses elicited by the scenarios affect sunk-cost scores. Study 3 replicated the findings in previous experiments, and additionally experimentally demonstrated that the relation between sunk-cost scores and affective reaction is moderated by justification; Participants who justified their decision were more resistant to the sunk-cost fallacy and showed less negative affect elicited by the scenarios, than participants who did not justify their decision. Study 4 again replicated the findings of the previous Studies, and additionally demonstrated that the relation between affective reaction and the sunk-cost fallacy is more prominent when participants are under high cognitive load than when under low load. Studies 3 & 4 demonstrated that the experience of affect as a function of sunk-cost scenarios is moderated by justification and cognitive load.

Our findings are in line with Wong and Kwong [16] who investigated the role of anticipated regret in committing the sunk-cost fallacy. They demonstrated that commitment to the fallacy is stronger when the possibility of future regret about withdrawal of commitment is high, than when this possibility is low. Besides the effect of anticipated regret about *withdrawal of commitment*, they showed an opposite effect for regret about *persistence of commitment* in escalating situations. I.e., Wong and Kwong [16] found both positive and negative relationships between anticipated regret and escalation, depending on the target of the emotion (withdrawal or persistence). Presumably, thinking over the likelihood of experiencing regret after discontinuing (or after continuing) investing induces a negative affective reaction, which in turn leads to commitment to the sunk-cost fallacy (or persistence to the escalating situation). It is important to be cautious when comparing our findings with that of Wong and Kwong [16]; The scenario contexts were fundamentally different in both studies. Hence, the findings in the two studies cannot be directly compared and thus were not contradictory.

Research demonstrated that judgment and decision-making can be affected by both types of affect (incidental affect: e.g., [23]; integral affect: e.g., [22]). Some researchers argued that incidental and integral affect work in unison to produce one affective feeling that guide judgment and decision-making ([45], p.88; [46], p.727; [47]).

As mentioned in the introduction, the link between the sunk-cost fallacy and affect has been demonstrated by other researchers. Their studies focused mostly on effects of incidental emotional states mostly (e.g, trait anxiety, neuroticism, induced anger and fear, reduced state negative affect). The reported studies in this article, in contrast, focused on the experience of integral affect. That is, affect elicited by each scenario. This suggests there are other ways to be shielded against the sunk-cost fallacy than affecting incidental affect. Studies 3 & 4 demonstrated that the sunk-cost effect is not only mediated by the experience of integral affect, but is also moderated by methods in which the course of action in sunk-cost scenarios is decided; Both Studies 3 & 4 showed that generating justification and reducing cognitive load can shield us against the sunk-cost fallacy.

As illustrated above, a variety of studies examined the role of affect in the sunk-cost fallacy. Conclusions of these studies were, however, not all in line with each other. Studies that examined trait anxiety, anger, and state negative affect, demonstrated, and concluded, that negative affect was related to employment of the fallacy. Studies on post-escalation and anticipated regret, depression, and neuroticism, however, found a negative relationship between negative affect and the sunk-cost fallacy. These findings underline the importance to consider, and to tear apart the role of, qualitative characteristics of different forms of affective reaction when examining the sunk-cost fallacy.

In their paper, Arkes and Blumer [1] compared theories on employment of the sunk-cost fallacy, and cognitive dissonance theory (CDT: [48]). According to both CDT and in theories on employment of the sunk-cost fallacy, a person continues investing resources when resources are already invested (CDT equivalent; improve attitude toward the task), compared to situations when no prior investment has been made. However, the motivation for this behavior is different for both phenomena: According to Festinger [48], dissonance in CDT occurs when there are inconsistencies between certain beliefs, opinions, or behaviors one have. This inconstancy produces discomfort and, correspondingly, pressures arises to reduce or eliminate this dissonance. This can, among other ways, be accomplished by changing views or behavior (e.g., continuing investing in a failing plan). As noted in the introduction, theorizing by other authors suggested that the sunk-cost effect is driven by negative feelings caused by the prospect of having invested without success. Arkes and Blumer [1] also noted differences between the two theories; People who lack sufficient justification for their action improve their evaluation of the task according to CDT. In sunk-cost situations, however, it is unlikely that people experience positive feelings when lacking justification. Indeed, Study 3 not only replicated Fennema and Perkins [49] who demonstrated moderating effects of justification on employment of the sunk-cost fallacy, but also showed that participants who justified their decision reported a more positive affective reaction, than decisions that were not justified.

In a recent paper, Feldman and Wong [50] offered a new perspective on the classic escalation-of-commitment phenomenon, which encompasses sunk-costs, negative-feedback, and a decision between escalation and de-escalation [51]. The authors argued that negative-feedback results in the tendency to take action, regardless of what that action may be. They demonstrated that framing escalation as action and de-escalation as inaction resulted in a stronger tendency to escalate than framing de-escalation as action and escalation as inaction. Landman [52] demonstrated that action elicit stronger affective reactions (positive after successful acts, and negative affective reaction over unsuccessful acts) than non-action.

Given that action (vs. in-action) resulted in stronger sunk-cost effects, and stronger affective reactions, in response to sunk-cost scenarios, provided supporting evidence for our finding that the sunk-cost effect is caused by integral affect, elicited by the specific decision context.

To conclude, our research shows that the negative integral affect that individuals experienced in the sunk-cost decision context deter them from withdrawal from the commitment, possibly because individuals tend to avoid taking actions when experiencing negative affect. As a result, they keep investing in a failing plan. We further found that asking individuals to justify their decision or avoiding depleting their mental resources are possible ways to alleviate the negative integral affect, the key mediator, thereby shielding individuals from committing the sunk-cost fallacy.

## Appendix A

Sunk-cost scenarios Study 1 (* scenarios selected for Study 3; [28]).

### Scenario 1

You are buying a gold ring on layaway for someone special. It costs $200 and you have already paid $100 on it, so you owe another $100. One day, you see in the paper that a new jewelry store is selling the same ring for only $90 as a special sale, and you can pay for it using layaway. The new store is across the street from the old one. If you decide to get the ring from the new store, you will not be able to get your money back from the old store, but you would save $10 overall.

Would you be more likely to continue paying at the old store or buy from the new store?

$$\begin{array}{c} 1\;2\;3\;4\;5\;6\\ \mathrm{Most}\;\mathrm{likely}\;\mathrm{to}\;\mathrm{Most}\;\mathrm{likely}\;\mathrm{to}\\ \mathrm{continue}\;\mathrm{paying}\;\mathrm{at}\;\mathrm{the}\;\mathrm{old}\;\mathrm{store}\;\mathrm{buy}\;\mathrm{from}\;\mathrm{the}\;\mathrm{new}\;\mathrm{store} \end{array}$$

### Scenario 2

You enjoy playing tennis, but you really love bowling. You just became a member of a tennis club, and of a bowling club, both at the same time. The membership to your tennis club costs $200 per year and the membership to your bowling club $50 per year. During the first week of both memberships, you develop an elbow injury. It is painful to play either tennis or bowling. Your doctor tells you that the pain will continue for about a year.

Would you be more likely to play tennis or bowling in the next six months?

$$\begin{array}{c} 1\;2\;3\;4\;5\;6\\ \mathrm{Most}\;\mathrm{likely}\;\mathrm{to}\;\mathrm{Most}\;\mathrm{likely}\;\mathrm{to}\\ \mathrm{play}\;\mathrm{tennis}\;\mathrm{play}\;\mathrm{bowling} \end{array}$$

### Scenario 3

You have been looking forward to this year’s Halloween party. You have the right cape, the right wig, and the right hat. All week, you have been trying to perfect the outfit by cutting out a large number of tiny stars to glue to the cape and the hat, and you still need to glue them on. On the day of Halloween, you decide that the outfit looks better without all these stars you have worked so hard on.

Would you be more likely to wear the stars or go without?

$$\begin{array}{c} 1\;2\;3\;4\;5\;6\\ \mathrm{Most}\;\mathrm{likely}\;\mathrm{to}\;\mathrm{Most}\;\mathrm{likely}\;\mathrm{to}\\ \mathrm{wear}\;\mathrm{stars}\;\mathrm{not}\;\mathrm{wear}\;\mathrm{stars} \end{array}$$

### Scenario 4

After a large meal at a restaurant, you order a big dessert with chocolate and ice cream. After a few bites you find you are full and you would rather not eat any more of it.

Would you be more likely to eat more or to stop eating it?

$$\begin{array}{c} 1\;2\;3\;4\;5\;6\\ \mathrm{Most}\;\mathrm{likely}\;\mathrm{to}\;\mathrm{Most}\;\mathrm{likely}\;\mathrm{to}\\ \mathrm{eat}\;\mathrm{more}\;\mathrm{stop}\;\mathrm{eating} \end{array}$$

### Scenario 5*

You are in a hotel room for one night and you have paid $6.95 to watch a movie on pay TV. Then you discover that there is a movie you would much rather like to see on one of the free cable TV channels. You only have time to watch one of the two movies.

Would you be more likely to watch the movie on pay TV or on the free cable channel?

$$\begin{array}{c} 1\;2\;3\;4\;5\;6\\ \mathrm{Most}\;\mathrm{likely}\;\mathrm{to}\;\mathrm{Most}\;\mathrm{likely}\;\mathrm{to}\\ \mathrm{watch}\;\mathrm{pay}\;\mathrm{TV}\;\mathrm{watch}\;\mathrm{free}\;\mathrm{cable} \end{array}$$

### Scenario 6*

You have been asked to give a toast at your friend’s wedding. You have worked for hours on this one story about you and your friend taking drivers’ education, but you still have some work to do on it. Then you realize that you could finish writing the speech faster if you start over and tell the funnier story about the dance lessons you took together.

Would you be more likely to finish the toast about driving or rewrite it to be about dancing?

$$\begin{array}{c} 1\;2\;3\;4\;5\;6\\ \mathrm{Most}\;\mathrm{likely}\;\mathrm{to}\;\mathrm{Most}\;\mathrm{likely}\;\mathrm{to}\\ \mathrm{write}\;\mathrm{about}\;\mathrm{driving}\;\mathrm{write}\;\mathrm{about}\;\mathrm{dancing} \end{array}$$

### Scenario 7*

You decide to learn to play a musical instrument. After you buy an expensive cello, you find you are no longer interested. Your neighbor is moving and you are excited that she is leaving you her old guitar, for free. You’d like to learn how to play it.

Would you be more likely to practice the cello or the guitar?

$$\begin{array}{c} 1\;2\;3\;4\;5\;6\\ \mathrm{Most}\;\mathrm{likely}\;\mathrm{to}\;\mathrm{Most}\;\mathrm{likely}\;\mathrm{to}\\ \mathrm{play}\;\mathrm{cello}\;\mathrm{play}\;\mathrm{guitar} \end{array}$$

### Scenario 8*

You and your friend are at a movie theater together. Both you and your friend are getting bored with the storyline. You’d hate to waste the money spent on the ticket, but you both feel that you would have a better time at the coffee shop next door. You could sneak out without other people noticing.

Would you be more likely to stay or to leave?

$$\begin{array}{c} 1\;2\;3\;4\;5\;6\\ \mathrm{Most}\;\mathrm{likely}\;\mathrm{to}\;\mathrm{Most}\;\mathrm{likely}\;\mathrm{to}\\ \mathrm{stay}\;\mathrm{leave} \end{array}$$

### Scenario 9

You and your friend have driven halfway to a resort. Both you and your friend feel sick. You both feel that you both would have a much better weekend at home. Your friend says it is "too bad" you already drove halfway, because you both would much rather spend the time at home. You agree.

Would you be more likely to drive on or turn back?

$$\begin{array}{c} 1\;2\;3\;4\;5\;6\\ \mathrm{Most}\;\mathrm{likely}\;\mathrm{to}\;\mathrm{Most}\;\mathrm{likely}\;\mathrm{to}\\ \mathrm{drive}\;\mathrm{on}\;\mathrm{turn}\;\mathrm{back} \end{array}$$

### Scenario 10*

You are painting your bedroom with a sponge pattern in your favorite color. It takes a long time to do. After you finish two of the four walls, you realize you would have preferred the solid color instead of the sponge pattern. You have enough paint left over to redo the entire room in the solid color. It would take you the same amount of time as finishing the sponge pattern on the two walls you have left.

Would you be more likely to finish the sponge pattern or to redo the room in the solid color?

$$\begin{array}{c} 1\;2\;3\;4\;5\;6\\ \mathrm{Most}\;\mathrm{likely}\;\mathrm{to}\;\mathrm{Most}\;\mathrm{likely}\;\mathrm{to}\\ \mathrm{finish}\;\mathrm{sponge}\;\mathrm{pattern}\;\mathrm{redo}\;\mathrm{with}\;a\;\mathrm{solid}\;\mathrm{color} \end{array}$$

## Appendix B

Two pairs of scenarios Study 2 & 4 [35].

### Scenario: Watching TV

*Vignette Version*: *Investment*

You are staying in a hotel room on vacation. You paid $10.95 to see a movie on pay TV. After 5 minutes, you are bored and the movie seems pretty bad. How much longer would you continue to watch the movie?

*Think about this situation as you normally would*. *Which of the following courses of action would you select*?

1. watch until the end
2. watch for 30 more minutes
3. watch for 20 more minutes
4. watch for 10 more minutes
5. stop watching entirely

*Vignette Version*: *Nonexistent or Smaller Investment*

You are staying in a hotel room on vacation. You turn on the TV and there is a movie on. After 5 minutes, you are bored and the movie seems pretty bad. How much longer would you continue to watch the movie?

*Think about this situation as you normally would*. *Which of the following courses of action would you select*?

1. watch until the end
2. watch for 30 more minutes
3. watch for 20 more minutes
4. watch for 10 more minutes
5. stop watching entirely

### Scenario: Working on project

*Vignette Version*: *Investment*

You have been working on a project related to one of your hobbies for five years. Lately, you have lost interest in the project. Whenever you work on the project, you are bored and wish that you were doing something else.

*Think about this situation as you normally would*. *Which of the following courses of action would you select*?

1. remain committed to the project
2. wait for six months to see if interest in the project increases
3. wait for a month or two to see if interest in the project increases
4. wait for a couple of weeks to see if interest in the project increases
5. stop working on the project immediately

*Vignette Version*: *Nonexistent or Smaller Investment*

You have been working on a project related to one of your hobbies for the past month. Lately, you have lost interest in the project. Whenever you work on the project, you are bored and wish that you were doing something else.

*Think about this situation as you normally would*. *Which of the following courses of action would you select*?

1. remain committed to the project
2. wait for six months to see if interest in the project increases
3. wait for a month or two to see if interest in the project increases
4. wait for a couple of weeks to see if interest in the project increases
5. stop working on the project immediately

## Supporting information

S1 File SPSS Data Study 1. (SAV)

S2 File SPSS Syntax Study 1. (SPS)

S3 File SPSS Data Study 2. (SAV)

S4 File SPSS Syntax Study 2. (SPS)

S5 File SPSS Data Study 3. (SAV)

S6 File SPSS Syntax Study 3. (SPS)

S7 File SPSS Data Study 4. (SAV)

S8 File SPSS Syntax Study 4. (SPS)
